# A systematic review on omics data (metagenomics, metatranscriptomics, and metabolomics) in the role of microbiome in gallbladder disease

**DOI:** 10.3389/fphys.2022.888233

**Published:** 2022-08-30

**Authors:** Paola Di Carlo, Nicola Serra, Rosa Alduina, Riccardo Guarino, Antonio Craxì, Anna Giammanco, Teresa Fasciana, Antonio Cascio, Consolato M. Sergi

**Affiliations:** ^1^ Department of Health Promotion, Maternal-Childhood, Internal Medicine of Excellence G. D’Alessandro, Section of Infectious Disease, University of Palermo, Palermo, Italy; ^2^ Department of Public Health, University “Federico II”, Naples, Italy; ^3^ Department of Biological, Chemical and Pharmaceutical Sciences and Technologies (STEBICEF), University of Palermo, Palermo, Italy; ^4^ Department of Health Promotion, Maternal-Childhood, Internal Medicine of Excellence G. D’Alessandro, Section of Gastroenterology, University of Palermo, Palermo, Italy; ^5^ Department of Health Promotion, Maternal-Childhood, Internal Medicine of Excellence G. D’Alessandro, Section of Microbiology, University of Palermo, Palermo, Italy; ^6^ Children’s Hospital of Eastern Ontario (CHEO), University of Ottawa, Ottawa, ON, Canada; ^7^ Department of Pediatrics, Stollery Children’s Hospital, University of Alberta, Edmonton, AB, Canada

**Keywords:** gallbladder disease, bile, human microbiota, taxonomy, cancer

## Abstract

Microbiotas are the range of microorganisms (mainly bacteria and fungi) colonizing multicellular, macroscopic organisms. They are crucial for several metabolic functions affecting the health of the host. However, difficulties hamper the investigation of microbiota composition in cultivating microorganisms in standard growth media. For this reason, our knowledge of microbiota can benefit from the analysis of microbial macromolecules (DNA, transcripts, proteins, or by-products) present in various samples collected from the host. Various omics technologies are used to obtain different data. Metagenomics provides a taxonomical profile of the sample. It can also be used to obtain potential functional information. At the same time, metatranscriptomics can characterize members of a microbiome responsible for specific functions and elucidate genes that drive the microbiotas relationship with its host. Thus, while microbiota refers to microorganisms living in a determined environment (taxonomy of microorganisms identified), microbiome refers to the microorganisms and their genes living in a determined environment and, of course, metagenomics focuses on the genes and collective functions of identified microorganisms. Metabolomics completes this framework by determining the metabolite fluxes and the products released into the environment. The gallbladder is a sac localized under the liver in the human body and is difficult to access for bile and tissue sampling. It concentrates the bile produced in the hepatocytes, which drains into bile canaliculi. Bile promotes fat digestion and is released from the gallbladder into the upper small intestine in response to food. Considered sterile originally, recent data indicate that bile microbiota is associated with the biliary tract’s inflammation and carcinogenesis. The sample size is relevant for omic studies of rare diseases, such as gallbladder carcinoma. Although in its infancy, the study of the biliary microbiota has begun taking advantage of several omics strategies, mainly based on metagenomics, metabolomics, and mouse models. Here, we show that omics analyses from the literature may provide a more comprehensive image of the biliary microbiota. We review studies performed in this environmental niche and focus on network-based approaches for integrative studies.

## Introduction

The advent of the Omics Sciences, based on Genomics, Transcriptomics, Proteomics, and Metabolomics, enabled the study of the human microbiota from another perspective, leading to the discovery of a connection between microbiota and the health of the host and identifying the characteristics of the microbiome that can contribute to diseases (Marchesi and Ravel, 2015).

The intestinal microbiota is a system composed of microorganisms that settle in the gastrointestinal tract and preserve physiological and metabolic well-being. Particular attention in recent years has been paid to the study of bacterial colonization of the gastrointestinal tract. It has been demonstrated that some bacteria are associated with the development of cancer disease (de Almeida et al., 2018; Maekawa et al., 2018; Mendez et al., 2020; Nokhandani et al., 2021; Saab et al., 2021). A multi-omics approach may help elucidate the gut microbiota activity and show that selected intestinal bacterial communities could play a role in developing chronic inflammatory disorders and neoplasms. In this regard, the composition of the biliary microbiota has attracted the interest of research aimed at clarifying the role of specific players in stimulating chronic inflammation and producing carcinogenic metabolites in the biliary tract, gallbladder, and pancreas (Molinero et al., 2019; Mendez et al., 2020; Visekruna and Luu, 2021).

The gallbladder may be affected by numerous disorders ranging from infectious processes to chronic inflammatory diseases and cancer (Apstein and Carey, 1996; Ebata et al., 2016). Since the gallbladder is a small pouch that collects bile produced by the liver, a change in bile composition, especially in cholesterol, is responsible for cholelithiasis. In 1967, Drs. Scott and Khan, two scientists of the Royal Free Hospital, London, United Kingdom (Scott and Khan, 1967), published a seminal work that showed the presence of bacteria in the bile of subjects undergoing uncomplicated cholecystectomy. Subsequently, about 20 years later, Wells and others highlighted the concept that bile is not genuinely sterile. Its colonization may represent a risk factor for postoperative sepsis concerning patients undergoing biliary tract surgery (Wells et al., 1989). The biliary system is in continuous contact with the gut bacteria. In fact, microorganisms ascend from the intestinal tract to the gallbladder, and gut microbial products influence bile salt metabolism (Small, 2003; Ebata et al., 2016). Currently, the term human bile microbiota refers to the microbial ecosystem of the gallbladder (Ebata et al., 2016). Studies suggest that the microbiota may substantially influence the outcome of gallbladder disorders (van Velkinburgh and Gunn, 1999; Prouty and Gunn, 2000; Bina and Mekalanos, 2001; Sleator et al., 2005; Torres et al., 2007; Crawford et al., 2008). Bile microbiota composition may show *Salmonella* spp. and other enteric commensals and pathogens, such as *Vibrio cholera*, *Campylobacter jejuni*, *Escherichia coli*, and *Listeria monocytogenes.* The latter have also demonstrated their ability to survive in the biliary environment, even though bile salts display antimicrobial properties (Begley et al., 2005; Hardy et al., 2006; Hung et al., 2006).

Variations within the human microbiota, such as an imbalance in bacterial composition, changes in bacterial metabolic activities, or changes in bacterial distribution, are defined as *dysbiosis* (Ebata et al., 2016). The term *gallbladder dysbiosis* has become the subject of revived attention in both human and experimental animals (Ebata et al., 2016; Wang et al., 2017; Gutierrez-Diaz et al., 2018; Xu et al., 2018). Changes in the microbial composition can cause a dire imbalance favoring pathogenic bacteria. They can lead to inflammation that contributes to different diseases, such as obesity, diabetes mellitus, virgola, multiple sclerosis, and cancer. Studies on bacterial bile composition have detected microorganisms with a possible molecular biologic role in chronic inflammation and carcinogenesis (Nath et al., 2010; Koshiol et al., 2016; Sharma et al., 2017). Investigations on the culture of bile samples collected by endoscopic retrograde cholangiopancreatography (ERCP) have identified a pattern of bacteria isolated in patients with diseases of the biliary tract, gallbladder, and pancreas impacting the survival of our patients affected with cancer (Di Carlo et al., 2018; Serra et al., 2018; Di Carlo et al., 2019; Sergi et al., 2019; Serra et al., 2021). On the other hand, other bile components, such as cholesterol and its derivates, have been investigated by biochemistry. This data involves microbial activities and cholesterol in the genesis of benign and malignant gallbladder disorders (Crawford et al., 2008; Nath et al., 2010; Phelan et al., 2017; Sergi et al., 2019; Kiss et al., 2020; Gruner and Mattner, 2021).

To overcome the drawbacks encountered in a culture-dependent approach, the microbiota study may exploit the progress of the most recent omics technologies, computational analytics, and deep neural network applications (Milani et al., 2021). Currently, the microbial composition of an organ or a niche can be explored by detecting the genetic material of microbes through the next-generation sequencing (NGS) of conserved microbial genes (microbiome analysis) using the metagenomic DNA extracted from the whole sample. Metagenomic DNA can also be used for Whole Genome Sequencing (WGS) to obtain data on the genetic background of the microorganisms present in the sample (Cho and Blaser, 2012; Liu et al., 2012; Armour et al., 2019). Furthermore, metagenomics allows us to investigate the metataxonomic and functional profiles through the shotgun approach (Milani et al., 2021). Metagenomics can quickly provide data on different microbial communities in healthy individuals and subjects suffering from various diseases (Armour et al., 2019; Xing et al., 2019; Little et al., 2020; Galluzzo et al., 2021; Saab et al., 2021). As for other disorders, the advances in sequencing technologies and computational methods are facilitating the investigation of the gallbladder microbiotas and serving in the diagnostic and therapeutical approaches to benign and malignant tumors (Hsing et al., 2007).

The method of metatranscriptomic provides a snapshot of gene expression in a given sample at a given time under specific conditions by capturing the total mRNA. However, it is technically an arduous study if it has to be thorough because RNA samples are more challenging to handle than DNA samples.

Environmental conditions can also influence the composition of microorganisms. For example, in the case of the gallbladder, variations in bile composition, especially bile salt and fats (i.e., cholesterol, fatty acids, and lecithin), may affect the microbiome (Small, 2003; Boyer and Soroka, 2021). Metatranscriptomics could help understand the interactions between the biliary components, especially the bile acids of the lipid component, and the microbial species that are part of the microbial community of the gallbladder (Botero et al., 2005; Carvalhais et al., 2012; Aguiar-Pulido et al., 2016; de Vos et al., 2022). Metatranscriptomics, metaproteomics, and metabolomics are powerful integrative approaches to metagenomics, shaping precision medicine, i.e., the medical model that recommends the customization of healthcare with medical decisions and therapies being tailored to a subgroup of patients. In addition, omics technologies expose microbial activity and interaction, enabling a better understanding of the interplay between the microbial community and the environment (Aw and Fukuda, 2015; Ye et al., 2016; Armour et al., 2019; Milani et al., 2021). However, these studies may struggle to recruit a large number of patients. Therefore, multicenter studies and the establishment of biorepositories are critical for these investigations (Sergi, 2022a).

This review focuses on describing the current state of gallbladder disorders with multiple approaches, including metagenomics, metatranscriptomics, and metabolomics. This review aims to summarize several studies to increase available knowledge of the connection between biliary microbiota and gallbladder disease.

### Choice of the literature on the role of microbiotas in gallbladder disease

The review process was conducted by identifying the research problem, performing bibliographic research, and conducting data evaluation and interpretation. The study was conducted using the PubMed electronic database. The formulation of search terms/keywords and research on electronic databases was performed by two researchers with degrees in Biostatistics and Epidemiology to ensure greater validity and reduce biases. The limitations put into place as filters were the human population, English language, time range (January 2015—December 2020), and scientific articles where the bile microbiota analysis was approached using Omics methodologies. In addition, studies such as preclinical studies, validation studies, meta-analyses, systematic reviews, and studies involving pediatric and neonatal patients were excluded.

Selection criteria included articles from national and international scientific literature whose title and content contained at least one of the keywords or a link to them. After carefully reading the abstract, the selection was made, and only studies that met the previously described inclusion and exclusion criteria were selected. The full texts were evaluated according to the same inclusion/exclusion criteria for all selected articles to identify those eligible for review.

Table 1 provides the selection criteria and the search string adopted in PubMed. Thirty-one articles published between 2015 and 2020 were identified through the database search. Particularly in this study we did not consider the papers published in 2021 apart from two papers published online in October 2020. Therefore papers such as Zhai W. et al., 2021 and Wei B, et al. (2021) were considered because published online and indexed in PubMed in 2020.

**TABLE 1:** Article selection criteria.


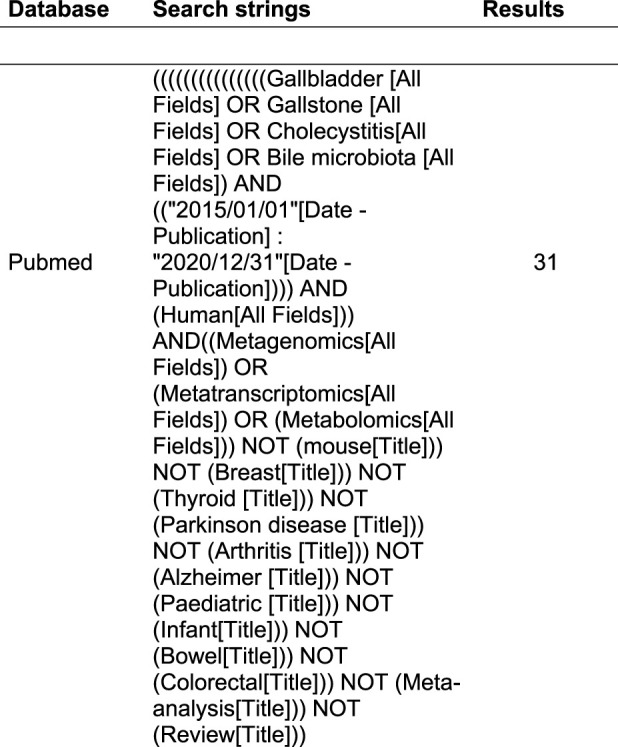

**FIGURE 1:**
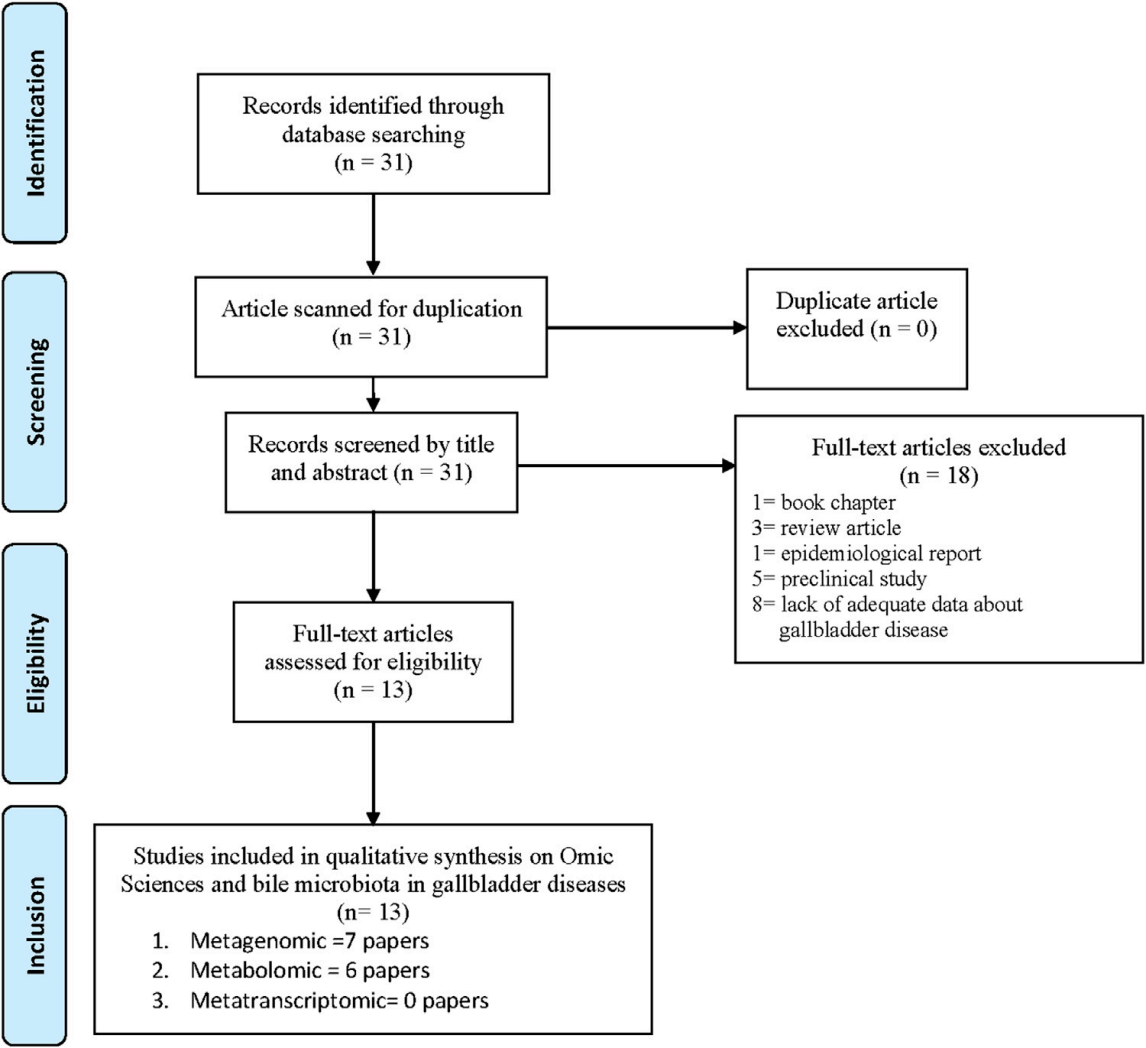
PRISMA Flow Chart showing the selected Papers.

Overall, the PRISMA flow chart diagram steps are depicted in Figure 1, summarizing the literature review process. Eighteen papers did not fit all our inclusion criteria and were excluded. In particular, the following contributions were excluded: one chapter book (Ranjan and Sinha, 2019a), three reviews (Wang et al., 2018; Ranjan and Sinha, 2019b; Nicoletti et al., 2020), one epidemiological report (Weaver et al., 2020), five preclinical studies (Gu et al., 2015; Liu et al., 2017; Kuerbanjiang et al., 2018; Kumar et al., 2018; Villar-Lorenzo et al., 2019), and eight papers that included patients with disorders other than gallbladder disease (Ferslew et al., 2015; Takis et al., 2018; Huang et al., 2019; Kang et al., 2019; Machado et al., 2020; Simeoli et al., 2020; Kulterer et al., 2021; Shao et al., 2021).

Thirteen selected articles met the inclusion criteria and were discussed. They are summarized in Table 2 (Shen et al., 2015; Stepien et al., 2016; Sharma et al., 2017; Kose et al., 2018; Näsström et al., 2018; Tsuchiya et al., 2018; Mohajeri et al., 2019; Molinero et al., 2019; Lee et al., 2020; Petrov et al., 2020; Song et al., 2020; Wei et al., 2021; Zhang et al., 2021). In particular, seven studies focus on metagenomic technologies and gallbladder disorders (Shen et al., 2015; Kujiraoka et al., 2017; Kose et al., 2018; Molinero et al., 2019; Lee et al., 2020; Song et al., 2020; Wei et al., 2021) and six others on metabolomic technologies and gallbladder disorders (Stepien et al., 2016; Sharma et al., 2017; Näsström et al., 2018; Mohajeri et al., 2019; Ranjan and Sinha, 2019a; Petrov et al., 2020; Zhang et al., 2021). The article by Molinero et al. analyzed gallbladder disease via metagenomic and metabolomic technologies (Molinero et al., 2019). No metatranscriptomics study on gallbladder disorders was found in the selected time range.

**TABLE 2:** Studies of the current review.

Title, first author, study year	Sample	Subjects and/or gallbladder disorder	Omics technology	Goal of study	Conclusion
Metagenomic sequencing of bile from gallstone patients to identify different microbial community patterns and novel biliary bacteria Shen et al. (2015)	Bile, oral respiratory and fecal sample	N Total pts = 15 Choledocholithiasis (CL) (*n* = 15)	WMS and 16S sequencing and bacterial oxidative stress responses	Bacteria involving in gallstones disease	Oral cavity and respiratory tract inhabitants were more prevalent in bile samples than intestinal inhabitants
Alteration of amino acid and biogenic amine metabolism in hepatobiliary cancers: Findings from a prospective cohort study Stepien et al. (2016)	Blood	N Total pts = 324 hepatocellular carcinoma (HCC) (*n* = 147), intrahepatic bile duct cancer (*n* = 43), gallbladder tract cancer (GBTC cases^a^) (*n* = 134)	Liquid chromatography (UPLC) coupled to a Q-Trap mass spectrometer	21 standard AA, 6 biogenic amines and hexoses	No significant associations of AA levels were found with risk of IHBC or GBTC.
H nuclear magnetic resonance (NMR)-based serum metabolomics of human gallbladder inflammation Sharma et al. (2017)	Blood	N Total pts = 71 Chronic Cholecystitis (CC) (*n* = 41) versus control group (*n* = 30)	Nuclear magnetic resonance spectroscopy (H NMR, spectra)	11 metabolites, alanine, formate, 1,2-propanediol, lipid, acetate, glutamine, histidine, lactate, glutamate, tyrosine, and histidine	Glutamine and glutamate, pyruvate, glyoxylate and dicarboxylate, histidine and alanine aspartate glutamate pathways are altered in CC
Metagenomics of pigmented and cholesterol gallstones: the putative role of bacteria Kose et al. (2018)	Choledocholithiasis	N total pts = 2 (pilot study)	Metagenomic by V4 region of bacterial 16S rRNA, functional metagenomic profile, stress analysis and biofilm formation	Potential role in gallstone formation	ORFs/promotors/proteins induced by bile showed biofilm due to *Klebsiella*. *E. coli*, *Enterobacter*, *Serratia*, *Shigella* (gram negative), *Enterococcus* and *Bacillus* (gram positive)
Diagnostic metabolite biomarkers of chronic typhoid carriage Näsström et al. (2018)	Blood	N Total pts = 37 *Salmonella Typhi* (*n* = 12) or *Salmonella Paratyphi A (n= 5)* gallbladder human carriage and non-carriage controls (*n* = 20)	2D-gas chromatography coupled with time-of-flight mass spectrometry (GCxGC-TOFMS)	195 metabolites generated from GCxGC-TOFMS analysis of plasma samples from patients in Nepal undergoing cholecystectomy	Five metabolites after comparing metabolite patterns obtained during chronic *Salmonella* carriage and acute enteric fever, respectively, could significantly distinguish *Salmonella* carriers from non-carriers
Metagenomics of Microbial Communities in Gallbladder Bile from Patients with Gallbladder Cancer (GBL) or Cholelithiasis (CL) Tsuchiya et al. (2018)	Bile	N Total pts = 37 GBL (*n* = 7) versus CL (*n* = 30)	Metagenomic by analysis of V3-V4 hypervariable regions of 16S rDNA	Microbiota in GBC versus cholelithiasis	*Fusobacterium nucleatum*, *E. coli*, and *Enterobacter spp*. in GBC; *E. coli*, *Salmonella spp*., and *Enterococcus gallinarum* in CL
*In vivo* H MRS of human gallbladder bile in understanding the pathophysiology of primary sclerosing cholangitis (PSC): Immune-mediated disease versus bile acid-induced injury Mohajeri et al. (2019)	Bile	N Total pts = 24 PSC (*n* = 10) versus healthy controls (*n* = 14)	H NMR spectra	BAs, cholesterol, glycine-conjugated bile acids, taurine-conjugated bile acids, and choline containing phospholipids	Statistically significant decrease in the levels of biliary metabolites in PSC versus controls
The human gallbladder microbiome is related to the physiological state and the biliary metabolic profile Molinero et al., 2019	Bile	N total pts = 27 Liver donors with previous antibiotic treatment (*n* = 13) and CL (*n* = 14)	Metagenomic by 16S rRNA gene of prokaryotic microorganisms and the 18S rRNA gene and metabolomic investigation by spectra analysis	Metagenomic and metabolomic investigation in healthy population and gallstones disease	Bacteroidaceae, Prevotellaceae, Porphyromonadaceae, and Veillonellaceae were more frequently detected. In CL pts difference of level and composition of acid salt in bile may be influenced by the gallstone genesis
16S rDNA microbiome composition pattern analysis as a diagnostic biomarker for biliary tract cancer Lee et al. (2020)	Blood	N total pts = 155 Biliary Tract Cancer (*n* = 24), cholangitis or cholecystitis (*n* = 43), and healthy controls (*n* = 88)	Microbiota composition by analysis of V3-V4 hypervariable regions of 16S rDNA	Microbiota composition in cholangitis, biliary tract cancer versus healthy population	The patients with inflammation and cancer had altered phyla composition in comparison to healthy group
Biliary Microbiota and Bile Acid Composition in Cholelithiasis (CL). Petrov et al. (2020)	Bile	N Total pts = 37 CL with (*n* = 21) and without *Opisthorchis felineus* infection (*n* = 16)	Liquid chromatography-mass spectrometry and tandem mass spectrometry	Primary Bas and its conjugates and secondary bile acids	Increase of taurocheno-deoxycholic acid and taurocholic acid concentration correlates with bile microbiota alpha-diversity. No differences in BA rates between *O. felineus*-infected and noninfected patients
A metagenomic study of biliary microbiome change along the cholecystitis-carcinoma sequence Song et al. (2020)	Mucosal biopsies	N total pts = 14 Chronic calculous cholecystitis (*n* = 7) versus Gallbladder cancer (GBC) (*n* = 7)	Illumina HiSeq × 10 platform (Illumina, Inc., United States)to sequence all samples and NCBI database for microorganism alignment	Composition of microbiota during Inflammation and cancer gallbladder disease	*Peptostreptococcus stomatis*, *Fusobacterium mortiferum*, and *Enterococcus faecium* were prevalent in GBC group
Analysis of bile acid profile in plasma to differentiate cholangiocarcinoma from benign biliary diseases and healthy controls^a^ Zhang et al. (2021) (Epub: 2020 October 28)	Blood	N total pts = 329 benign biliary diseases (BBD) (*n* = 120), Cholangiocarcinoma (CCA) (*n* = 42), gallbladder cancer (GBC) (*n* = 28), hepatocellular carcinoma (HCC) (*n* = 19), healthy controls (HC) (*n* = 120)	Mass spectrometry	To characterize the circulating BAs profile in CCA and BBD patients, as well as HC group, and to explore the potency and reliability of plasma BAs as biomarkers for CCA diagnosis	Specific changes in plasma concentrations of BAs may serve as diagnostic biomarkers for distinguishing CCA from BBD and HC
Alterations of gut microbiome in patients with type 2 diabetes mellitus (T2DM) with and without cholecystectomy Wei et al. (2021) (Epub: 2020 November 9)	Stool	N total pts = 56 new-onset (T2DI = 21pts) and long-term T2DM (T2DII = 21 pts) with cholecystectomy versus T2DM without cholecystectomy (*n* pts = 14)	Microbiota composition by analysis of V3-V4 hypervariable regions of 16S rDNA	Cholecystectomy could partially alleviate long-term T2DM-induced dysbiosis	Cholecystectomy alleviated the increase in the Firmicutes abundance and increased the Fusobacteria abundance in long-term patients with T2DM.

**Notes**: H NMR, H nuclear magnetic resonance; BAs, biliary acids; BBD, benign biliary diseases, BTC, biliary tract cancer; healthy controls (HC); CC , chronic cholecystitis; CCA, cholangiocellular carcinoma; CL , cholelithiasis; HCC, hepatocellular carcinoma; GBC, gallbladder cancer; ^a^GBTC, includes tumours in the gallbladder, extrahepatic bile ducts, ampulla of Vater, and biliary tract; T2DM, type 2 diabetes mellitus; WMS, whole metagenome sequencing, or shotgun metagenome sequencing.

a The paper was published in PubMed in 2020.

### Metagenomic data

The human samples collected to characterize the gallbladder ecosystem, including bile and tissue sampling, were heterogeneous regarding the metagenomic articles. Song et al.reported an investigation into mucosal biopsies of patients with gallbladder cancer (GBC) and chronic cholecystitis (CC) to find the pattern of microbiota: the goal of the study was to investigate the interaction between dysbiosis and inflammation in the genesis of cancer (Song et al., 2020). After mucosal DNA extraction, the authors identify different microbiotas found in the two groups of patients by using Illumina Next-Gene Sequencing ™ and NCBI database and metagenomic species analysis. The metagenomic species (MGS) profiling of the 25 most abundant bacterial species showed a significant difference in the oncologic patients with a prevalence of *Peptostreptococcus stomatitis*, *Fusobacterium mortiferum*, and *Enterococcus faecium*. Moreover, Song et al. analyzed how bacterial changes in the two groups of gallbladder disease may lead to changes in certain functional gene families, especially energy and carbohydrate metabolism. They hypothesized that this change could influence the development of gallbladder cancer in chronic cholecystitis (Song et al., 2020). The interpretation of the results in the other selected papers must consider that the genetic approaches to performing Whole Genome Sequencing (WGS) in metagenomic methodologies have been implemented by introducing metataxonomy, which uses amplicons from a targeted marker gene to make taxonomic inferences (Chaffron et al., 2010; Marchesi and Ravel, 2015). In the case of metataxonomics, reads are frequently grouped (or clustered) before assigning a label. One widespread marker gene used in metataxonomic studies is 16S rRNA. Groups of reads resulting from the clustering process and displaying similarity in sequence and/or composition are inferred to have a common origin and are referred to as operational taxonomic units (OTUs) (Chaffron et al., 2010). Lee et al. reported the microbiota composition through metagenomic analysis using the blood samples of healthy patients versus subjects with inflammation and cancer of the biliary tract. Overall, the patients with inflammation and/or cancer showed some differences in the percentage of family composition of Bifidobacteriaceae*,* Pseudomonaceae*,* Comamonadaceae, Oxalobacteraceae*,* and the *Corynebacterium* spp. Clostridia were prevalent in biliary tract cancer (Lee et al., 2020). Wei et al. (Wei et al., 2021) emphasized the role of dysbiosis in diabetic type 2 patients is influenced by gallstones disease and refined the effect of cholecystectomy on gut microbiota composition. Wei et al. found that *Fusobacterium* and *Bilophila* genera increased in diabetic patients who underwent cholecystectomy. Both genera characterized gut microbiota (Rinninella et al., 2019), and their role in inflammation and cancer of gut disorders is still under debate (Dahmus et al., 2018). In 2019, Molinero et al. (Molinero et al., 2019) analyzed the bile samples obtained from human liver donors during liver transplantation surgery. The metagenomic analysis of the biliary micro-ecosystem in individuals without hepatobiliary pathology showed that the main bacterial *phyla* were represented by Firmicutes, Bacteroidetes, Actinobacteria, and Proteobacteria. On the other hand, Bacteroidaceae, Prevotellaceae, Porphyromonadaceae, and Veillonellaceae were prevalent in patients suffering from choledocholithiasis. Kose et al. conducted a pilot study to elucidate the key factors in gallstone pathogenesis and formation via metagenomic analysis of cholesterol and pigmented gallstones collected from two patients (Kose et al., 2018). The microbial composition study showed an excellent biofilm production of the by-isolates. Moreover, these authors reported the complementary carbon and hydrogen isotopic analyses of cholesterol obtained after stress. The bacterial community of the pigmented stone resembled that of cholesterol gallstones, with *Klebsiella* spp., *Enterococcus* spp., *Enterobacter* spp., *E. coli*, and *P. aeruginosa* being the most prevalent genera. At the same time, bile resistance genes were also present in *Escherichia*, *Shigella*, *Serratia*, and *Bacillus* families. Furthermore, *Klebsiella* spp. was also present in one of the cholesterol gallstones. In contrast, the remaining cholesterol stones showed a predominance of Gram-positive bacteria not identified within the pigmented stones. Overall, *Klebsiella* spp. seem to be involved in biofilm formation. Therefore, the authors considered this pathogen a prominent microorganism in gallstone pathogenesis.

In 2018, Tsuchiya et al. compared the bacteria detected in bile samples of Bolivian and Chilean patients with GBC and cholelithiasis (CL) (Tsuchiya et al., 2018). This study showed bacterial infection rates in the bile of 42.9% in combined Bolivian and Chilean patients with GBC and 13.3% in patients with CL. The predominant species detected in Bolivian patients with GBC patients were *Fusobacterium nucleatum*. In Bolivian CL patients, *E. coli, Enterococcus gallinarum, and Salmonella* spp. were shown. In Chilean patients, the predominant species were *E. coli* and *Enterobacter* spp. in GBC patients and *E. coli* in the CL patients. Shen et al. compared the microbial communities of bile samples of Chinese patients affected by gallstone disease with the oral cavity and respiratory tract (Shen et al., 2015). These authors found that, besides typical intestinal microbes such as *Shigella* spp. and *Salmonella* spp., also oral cavity inhabitants (i.e., *Pyramidobacter piscolens, Raoultella ornithinolytica, Porphyromonas endodontalis*) and upper respiratory tract microorganisms (such as *Streptococcus* spp., *Neisseria* spp., *Prevotella* spp., *Veillonella* spp.) were identified in bile samples. The investigation by the gene predictions of bacterial products β-glucuronidase, phospholipase, and urease implicated in gallstone formation showed that seven bile samples had at least three species harboring genes *uidA* (encoding β-glucuronidase) and *pldA* (encoding phospholipase A1) related to gallstone formation.

Regarding the investigation of bile resistance, the sequence analysis of two mechanisms for bacterial bile resistance (bile salt deconjugation and multidrug efflux pump proteins) revealed a prevalence of genes encoding multidrug efflux pump proteins. It suggests that this might be a more favorable path to bacterial colonization and overgrowth in patients with gallstone disease. The gut microbiome of long-term type 2 diabetes mellitus (T2DM) patients who had undergone cholecystectomy and age- and/or sex-matched subjects of new-onset and long-term T2DM without cholecystectomy was assessed using 16S rRNA gene sequencing in stool samples (Wei et al., 2021). In this investigation, the gut microbiomes of long-term individuals with T2DM who received gall bladder resection (T2DIIC group) and age- and/or sex-matched individuals with new-onset (T2DI group) and long-term (T2DII group) T2DM without receiving cholecystectomy were thoroughly assessed. Firmicutes phylum and *Lachnospira genus* were increased in long-term patients with T2DM compared with T2DII subjects. Also, cholecystectomy increased the relative amount of the Fusobacteria and the *Fusobacterium* and *Bilophila* genera. In other words, the resection of the gallbladder may alleviate long-term DM-induced dysbiosis of the gut microbiota.

### Metabolomic data

In the articles related to the role of metabolomics technologies in human gallbladder disease, the authors quantify metabolites in samples of patients with gallbladder disease through technologies such as chromatography, mass spectrometry, and imaging using nuclear magnetic resonance (NMR). Thanks to these technologies, metabolomics can be considered a complete analysis. It is possible to characterize and quantify the metabolites detected in the biological fluid, i.e., serum, bile, and urine, in patients with gallbladder disease (Stepien et al., 2016; Sharma et al., 2017; Bellocchi et al., 2019; Mohajeri et al., 2019; Ranjan and Sinha, 2019a; Petrov et al., 2020). The metabolome is considered the most direct indicator of the health of an environment or any alterations of homeostasis (i.e., dysbiosis) since the variations of the metabolites can be linked to alterations of the metabolic pathways when there is a health alteration, such as inflammation or carcinogenic processes (Stepien et al., 2016; Ranjan and Sinha, 2019a; Ranjan and Sinha, 2019b). This methodology has numerous implications in studying the genesis of disorders and can help understand the influence of treatment on metabolic processes (de Almeida et al., 2018; Sinha et al., 2020). Moreover, this methodology might represent a step for the clinician to verify the health state of the human microbiota in the context of various diseases (Melis et al., 2021; Thapa et al., 2021).

Mohajeri et al. (Mohajeri et al., 2019), Molinero et al. (Molinero et al., 2019), Ranjan and Sinha (Ranjan and Sinha, 2019a), and Sharma et al. studied the metabolites using NMR spectroscopy (Sharma et al., 2017) (Table 2). Further aspects were investigated by Petrov et al. (Petrov et al., 2020), Zhang et al. (Zhang et al., 2021), Stepien et al. (Stepien et al., 2016), and Näsström et al. (Näsström et al., 2018). These authors studied the levels of amino acids and their derivatives and the biliary metabolites using mass spectrometry. In 2019, Molinero et al. investigated the difference between subjects with choledocholithiasis and healthy subjects using metagenomic and metabolomic methodologies (Molinero et al., 2019). In the metabolomic section, Molinero et al. analyzed the signals of bile acids using spectra methodology. This investigative report disclosed statistically significant differences between bile samples of patients with choledocholithiasis versus controls when examining the compounds of both the aromatic (glycine- and/or taurine-conjugated forms) and the aliphatic regions chenodeoxycholic (CDCA), deoxycholic acid (DCA), and cholic acids (CA) of bile acids (Molinero et al., 2019). In the contribution of Ranjan and Sinha, the authors reported the serum metabolic alterations in the chronic inflamed human gallbladder (CC) (Ranjan and Sinha, 2019a). In particular, the authors highlighted the fact that metabolite levels influenced the metabolic pathways and suggested that the alteration of metabolite levels in CC may have influenced a continuous progression to neoplasm (GBC). A functional interplay between bile acids and microbial composition was analyzed by Petrov et al. in bile samples obtained from gallstone disease patients, of which twenty-one with *Opisthorchis felineus* infection, during the laparoscopic cholecystectomy for gallstone disease in Siberian patients (Petrov et al., 2020). In this geographical area, the parasitic infection is endemic, and there were no differences in bile acid concentrations between *O. felineus*-infected and non-infected patients. These authors found a correlation between taurocholic acid (TCA), tauro-chenodeoxycholic acid (TCDCA), and alpha diversity of bile microbiota. In particular, they observed correlations between primary biliary acids (BAs) and bile bacteria, while fecal microbiota disturbance was associated chiefly with secondary BAs in feces.

Zhang et al. reported the results of a European prospective cohort study on the associations between blood levels of circulating amino acids (AA), biogenic amines, and hexoses panel and risks of developing hepatocellular carcinoma (HCC), intrahepatic bile duct cancer (IHBC), and biliary tract cancers (GBTC) (Zhang et al., 2021). A significant correlation was observed in all subjects between several AAs, biogenic amines, hexoses, and individual liver function biomarkers such as glutamine, glutamate, and gamma-glutamyltransferase (GGT). There was also a significant correlation with other liver function enzymes (aspartate aminotransferase, AST, alanine aminotransferase, ALT, and alpha-fetoprotein, AFP). Perturbations in levels of circulating AA metabolites were observed in HCC, but the data did not show any significant associations with IHBC or GBTC. These authors attributed the results obtained in patients with HCC to variable dosing of the metabolites in the blood samples. In fact, the liver is a highly metabolically active organ with a remarkable exposure to circulating metabolites.

The Mohajeri et al. study aimed to compare the bile metabolites in patients with primary sclerosing cholangitis (PSC) to those in healthy subjects (Mohajeri et al., 2019). The molar concentration of total bile acids (TBAs) (TBAs + cholesterol) was significantly lower in PSC patients than in healthy controls. The taurine-conjugated bile acids (TCBAs) levels were substantially lower in the PSC patients. In addition, choline-containing phospholipids were markedly lower in the PSC group. By analyzing metabolites in bile samples, Mohajeri et al. focused on which elements of bile composition might have caused damage in these patients. It is currently under debate whether changes in bile acid composition may influence the genesis of gallbladder disorders and/or their evolution.

Näsström et al. studied 195 selected metabolites using gas chromatography coupled with time-of-flight mass spectrometry in *Salmonella typhi* or *S. paratyphi A* human gallbladder carriage, and non-carriage controls (Näsström et al., 2018). The study data showed that five metabolites should be highlighted after comparing metabolite patterns obtained during chronic *Salmonella* carriage and acute enteric fever. They could significantly distinguish *Salmonella* carriers from non-carriers. Sharma et al. analyzed eleven selected metabolites in patients with CC versus a control group to better illuminate the role of metabolites in inflammation progress (Sharma et al., 2017). The analysis showed that the glutamine and glutamate, pyruvate, glyoxylate and dicarboxylate, histidine, and alanine aspartate glutamate pathways were altered in CC. Stepien et al. collected blood samples from 147 patients with HCC, 43 patients with IHBC, and 134 patients with GBTC (Stepien et al., 2016). The metabolites considered in this study included the standard amino acids and additional compounds, including creatinine, kynurenine, serotonin, and taurine hexoses. They also had elements of diet such as protein, carbohydrates, fat, alcohol, fibers, sugars, and energy. These authors refer to a perturbation in circulating AA metabolites detected in HCC but did not observe any significant associations between AA levels and risk of IHBC or GBTC.

## Discussion

Our review focuses on the role of omics’ technologies in identifying the changes in community biliary microbial composition or biliary dysbiosis (DeGruttola et al., 2016; Xing et al., 2019; Little et al., 2020; Saab et al., 2021) and biliary metabolites detected in the most common gallbladder disorders such as cholelithiasis, CC, GBC, and in patients with diabetes who underwent cholecystectomy (Ferslew et al., 2015; Shen et al., 2015; Stepien et al., 2016; Kujiraoka et al., 2017; Sharma et al., 2017; Kose et al., 2018; Näsström et al., 2018; Mohajeri et al., 2019; Kose et al., 2020). Animal-based studies showed a native biliary microbiota that changes in the course of gallbladder disease (Jimenez et al., 2014; Xing et al., 2019). It is not easy to study bile microbiota in healthy subjects for ethical reasons. Still, Molinero et al. collected bile samples from liver donors with no record of biliary or hepatic disorders and, via shotgun metagenomic analyses, corroborated the 16S rRNA gene data, detecting the existence of three main phyla - *Actinobacteria*, *Bacteroidetes*, *Firmicutes*-associated with some genera of the alpha division of *Proteobacteria* (Molinero et al., 2019). Moreover, these authors found in patients with cholelithiasis that *Bacteroides*, *Escherichia*, and *Shigella* were more abundant than in healthy subjects where the genera *Sphingomonas* (Proteobacteria) was abundant. Even though some enteric pathogens have a unique ability to resist the bactericidal effects of bile (Sistrunk et al., 2016), other bacteria can survive in bile thanks to bacterial gene encoding mechanisms of resistance to the biliary environment such as bile salt deconjugation and multidrug efflux pump proteins (Ruiz et al., 2013; Shen et al., 2015). Bile resistance-related genes, which could be crucial for bacterial survival, were identified by Shen *et al.* in patients with choledocholithiasis (Shen et al., 2015). These authors showed that in addition to the bacteria that make up the gut microbiota, other bacteria that colonize the oral cavity or upper respiratory tract could also become inhabitants of the biliary microbiota (Shen et al., 2015). This suggests that there is, indeed, a continuous change in the biliary microbiota.

The role of bacteria in gallstone formation is an old question (Cetta, 1991). Via omics technologies, bacterial genes associated with gallstones have been confirmed. Therefore, bacterial slime (i.e., glycocalyx), bacteria resistance in bile, and biofilm formation should play an essential role in gallstone formation (Shen et al., 2015; Kose et al., 2018; Molinero et al., 2019). It is well-known that extended periods of exposure to bile salts lead to biofilm formation among the enteric pathogens within the Enterobacteriaceae family. This concerns well-researched bacteria such as *Salmonella* and the *Shigella* species and other emerging pathogens including *E. coli*, *K. pneumoniae*, *Enterococcus* spp.*,* and *Clostridium* spp. (Begley et al., 2005; Hardy et al., 2006; Hung et al., 2006; Ye et al., 2016; Nickerson et al., 2017; Kose et al., 2018; Nickerson and Faherty, 2018; Mullish and Allegretti, 2021).

Biofilm formation and anaerobic energy metabolism are the potential microbial mechanisms of bacteria involved in gallstone formation (Kose et al., 2018). Kose et al. have studied the bacterial composition of stones and found enterobacteria such as *Klebsiellaspp*., *Escherichiaspp.*, *Enterococcusspp*., *Salmonellaspp.*, and *Enterobacter* spp. played a role in the formation of gallstones (Kose et al., 2018).

Carcinogenesis due to chronic inflammation caused by pathogen infections has been recognized as one of the carcinogenesis patterns in humans (Nokhandani et al., 2021). Song *et al.* performed metagenomic shotgun sequencing on mucosal biopsy samples collected in patients with CC and CL (i.e., cholecystitis accompanied by biliary stones) and GBC (Song et al., 2020). Song *et al.* showed that *Firmicutes*, *Bacteroidetes*, *Actinobacteria*, and *Proteobacteria* were detected in both groups. The authors analyzed the alpha diversity of the richness and uniformity of the species of the two groups during the development of GBC. They found differences in the composition of the biliary microbial community. *Peptostreptococcus stomatis*, *Fusobacterium mortiferum*, and *Enterococcus faecium* were present in more significant numbers in tissue samples of patients with GBC than those without neoplastic progression.

Moreover, to demonstrate a potential carcinogenic role in the different microbial compositions, these authors focused on the carbohydrate composition during the development of GBC. Bacterial bile detection via metagenomics in patients with GBC was studied in South America (Tsuchiya et al., 2018). Tsuchiya *et al.* reported a predominance of *Fusobacterium nucleatum*, *E. coli*, and *Enterobacter* spp. in Bolivian and Chilean patients with GBC (Tsuchiya et al., 2018). *Fusobacterium nucleatum*, a common component of the oral bacterial community, has recently emerged as a compelling candidate for causing human diseases given its prevalence in gut inflammation and cancer (Engevik et al., 2021).

In this review, we included the gut dysbiosis analysis in T2DM patients. They did not show a difference in *Fusobacterium* levels between the new-onset T2DM and long-termT2DM subjects, indicating that the duration of diabetes may not have a significant effect on *Fusobacterium*. On the other hand, the cholecystectomy significantly increased the amount of the *Fusobacterium* in long-term T2DM patients, which was reported to cause opportunistic infections or aggravate high-fat diet-induced metabolic disorders (Wei et al., 2021). Nevertheless, further studies, including a large sample and multicenter studies, may be needed to discriminate between physiology and pathology (Brennan and Garrett, 2019; Brennan et al., 2021).

Several scientific contributions mentioned the role of *Enterococcus* spp. in gallbladder disease (Shen et al., 2015; Kose et al., 2018; Tsuchiya et al., 2018; Molinero et al., 2019; Song et al., 2020). The role of *Enterococcus* spp. is under discussion because it has been detected in benign and malignant disorders of the gallbladder (Maekawa et al., 2018; Song et al., 2020). The disadvantage of *E. faecium* in bile and gut microbe composition is debated. The dangerous role of this bacterium has been proposed due to its ability to cause genomic DNA instability. There is also evidence that this microorganism predisposes the host to mutations toward carcinogenesis (de Almeida et al., 2018; Maekawa et al., 2018). In other studies, the multidrug-resistant Gram-negative pathogens and *Enterococcus* spp. were prevalent in biliary and pancreatic disorders (Di Carlo et al., 2018; Serra et al., 2018; Di Carlo et al., 2019; Sergi et al., 2019; Serra et al., 2021).

Metabolomics has focused on metabolites in serum or other body fluids, allowing the early diagnosis of gallbladder diseases. Consequently, it can help clinicians manage the gallbladder’s acute and chronic inflammatory processes. Näsström *et al.* identified metabolites that distinguish between infection and colonization due to *Salmonella typhi* or *S. paratyphi A* (Näsström et al., 2018). Sharma *et al.* analyzed eleven selected metabolites in patients with CC versus controls and showed that the metabolite dosage could identify the inflammation process and help to reduce its progress (Sharma et al., 2017). Similarly, Ranjan and Sinha stress that the serum dosage of specific metabolites in gallbladder inflammatory processes could help predict the risk of tumor evolution (Ranjan and Sinha, 2019a). Metabolomics might help us better understand the role of bile microbiota in cancer pathogenesis. In particular, Stepien *et al.* analyzed nutrition and specific AA in subjects with hepatobiliary cancer. Despite the authors’ efforts, it was challenging to affirm a correlation between the studied metabolites and cancer patients’ food habits. In conclusion, they used the term “perturbation” about the circulating AA metabolites dosage levels in HCC (Stepien et al., 2016). As indicated above, Zhang *et al.* underlined the difficulties of making an early diagnosis of biliary and gallbladder cancer compared to liver cancer because this organ is a particular metabolically active organ with constant exposure to circulating metabolites (Zhang et al., 2021). Associations between diversity, the taxonomic profile of bile microbiota, and bile BA levels were evidenced in patients with cholelithiasis (Petrov et al., 2020). Petrov *et al.* showed a correlation between primary biliary acids and bile microbiota composition (Petrov et al., 2020). At the same time, fecal microbiota dysbiosis was primarily associated with secondary BAs. All studies analyzing BAs focus on the harmful role of secondary BAs and their metabolites. The most significant interaction with the gut microbial community is probably converting primary BAs to secondary BAs (Ferslew et al., 2015; Mohajeri et al., 2019; Petrov et al., 2020; Sinha et al., 2020). Microorganisms in the bile are relevant only if we show that they activate the metabolic enzymatic process, modulating the primary and secondary BAs circuit.

Overall, the examination of the literature to write this review was not straightforward because the various methodologies were often heterogeneous. However, the articles in this review provide an array of research and a platform concerning the kind of human sample (serum, tissue, urine, e.g.), the heterogeneity of gallbladder disorders (cholelithiasis, cholecystitis, cancer, and patients with or without cholecystectomy), and the heterogeneity of analyzed elements (e.g., the composition of microbiota, BAs, and biliary metabolites), which may be useful for more congruent studies in the future. The human samples analyzed ranged from bile blood to non-blood tissue or other sites sampled using oral cavity or rectal swabs (Shen et al., 2015; Kujiraoka et al., 2017; Kose et al., 2018; Molinero et al., 2019; Kose et al., 2020; Lee et al., 2020; Song et al., 2020). It is noteworthy that metatranscriptomic studies in patients with gallbladder disease are missing. The lack of such studies is probably due to technical drawbacks in handling RNA molecules. In fact, mRNA is notoriously unstable, and sample integrity can be compromised before sequencing. In addition, distinguishing between host and microbial RNA can be challenging, although enrichment kits are now readily available on the market (Leimena et al., 2013; Mukherjee et al., 2017). Finally, much of the collected RNA comes from ribosomal RNA. Its dominant abundance can drastically reduce mRNA coverage (Mukherjee et al., 2017). Most metatranscriptomic studies focused on gut dysbiosis and the interplay of food intake and gut microbiota (Islam et al., 2011; Martinez et al., 2013; Rath et al., 2018; Franzosa et al., 2019). On this issue, metatranscriptomic studies conducted on laboratory animals such as mice or human fecal samples have recently shown that the harmful action of secondary BAs derives from primary BAs in the genesis of intestinal inflammatory processes and pancreatic cancer (Hildebrandt et al., 2009; Islam et al., 2011; Martinez et al., 2013; Zhang et al., 2016; Just et al., 2018). In addition, metatranscriptomic studies focused on RNA sequencing (RNA seq) also allow functional changes to be discovered to decipher what can affect antibiotic resistance gene expression after exposure to antibiotics. They can also study changes in the virulence factors of specific pathogens and epigenomic studies of some neoplastic conditions (Sharma et al., 2010; Korry et al., 2020; Auld et al., 2022).

In our systematic review, no article found *Candida* spp. in biliary *dysbiosis*. However, other authors have reported *Candida* spp. in biliary tract disorders, and *Candida albicans* is documented in the gut microbiome and gut *dysbiosis* (Rodolico et al., 2017; Gutierrez et al., 2020).

Current knowledge on the composition of the microbiota, its modifications concerning various physiological and pathological conditions, and the numerous mechanisms by which it can interact with the host have progressed rapidly in recent years. This aspect is due to the biotechnology pace over the last couple of years. The contribution of “omics” sciences, opening new perspectives on the role of the microbiota in the development of various systems and on the pathogenesis of many morbid conditions, has been remarkable in all fields of medicine. However, in many cases, the causal link between alterations of the bile microbiota and pathology remains to be consolidated, and the mechanisms that underlie it as it remains to strengthen the research field on the role of bile dysbiosis in rare conditions, such as gallbladder diseases.

Translating a newly developed methodology from the research laboratory to the clinical laboratory must consider analytical validity, clinical advantageousness, and financial responsibility. Therefore, using this approach in the routine clinical context and the implementation in laboratory information systems are not yet recommended (Sergi, 2022b). However, we need to stay tuned because it could be beneficial for identifying genera and species in the nearest future after validation and certification according to regulatory agencies, such as the College of American Pathologists.

## Conclusion

Overall, the recent development of omics approaches can globally quantify cellular changes at different molecular levels by combining data from multiple omics methodologies. These include the study of the genome, transcriptome, proteome, interactome, epigenome, metabolome, lipidome, and microbiome (Muller et al., 2013; Segata et al., 2013; Heintz-Buschart et al., 2016a; Heintz-Buschart et al., 2016b). This approach is called multi-omics and, in comparison to single omics, gathers information from multiple “omes” to better understand complex diseases (Heintz-Buschart et al., 2016a; Heintz-Buschart et al., 2016b; Kaysen et al., 2017; Lloyd-Price et al., 2019; Mendez et al., 2020). Future efforts are needed to make these new high-cost technologies available in treatment centers. The authors hope that this review will stimulate research and create a network of collaboration between research centers focusing on animal models, implementing biorepositories, and treatment centers for gallbladder diseases.

## Data Availability

The original contributions presented in the study are included in the article/supplementary materials, further inquiries can be directed to the corresponding author.
